# Circulating tumor cells exhibit stem cell characteristics in an orthotopic mouse model of colorectal cancer

**DOI:** 10.18632/oncotarget.8373

**Published:** 2016-03-25

**Authors:** Sebastian Schölch, Sebastián A. García, Naoki Iwata, Thomas Niemietz, Alexander M. Betzler, Lahiri K. Nanduri, Ulrich Bork, Christoph Kahlert, May-Linn Thepkaysone, Anka Swiersy, Markus W. Büchler, Christoph Reissfelder, Jürgen Weitz, Nuh N. Rahbari

**Affiliations:** ^1^ Department of Gastrointestinal, Thoracic and Vascular Surgery, Medizinische Fakultät Carl Gustav Carus, Technische Universität Dresden, Dresden, Germany; ^2^ Department of Gastroenterological Surgery, Nagoya University Graduate School of Medicine, Nagoya, Aichi, Japan; ^3^ Department of General, Gastrointestinal and Transplantation Surgery, University Hospital Heidelberg, Ruprecht-Karls-Universität Heidelberg, Heidelberg, Germany

**Keywords:** circulating tumor cells, colorectal cancer, mouse model, stem cells, metastasis

## Abstract

The prognosis of colorectal cancer (CRC) is closely linked to the occurrence of distant metastases, which putatively develop from circulating tumor cells (CTCs) shed into circulation by the tumor. As far more CTCs are shed than eventually metastases develop, only a small subfraction of CTCs harbor full tumorigenic potential. The aim of this study was to further characterize CRC-derived CTCs to eventually identify the clinically relevant subfraction of CTCs.

We established an orthotopic mouse model of CRC which reliably develops metastases and CTCs. We were able to culture the resulting CTCs *in vitro*, and demonstrated their tumor-forming capacity when re-injected into mice. The CTCs were then subjected to qPCR expression profiling, revealing downregulation of epithelial and proliferation markers. Genes associated with cell-cell adhesion (claudin-7, CD166) were significantly downregulated, indicating a more metastatic phenotype of CTCs compared to bulk tumor cells derived from hepatic metastases. The stem cell markers DLG7 and BMI1 were significantly upregulated in CTC, indicating a stem cell-like phenotype and increased capacity of tumor formation and self-renewal. In concert with their *in vitro* and *in vivo* tumorigenicity, these findings indicate stem cell properties of mouse-derived CTCs.

In conclusion, we developed an orthotopic mouse model of CRC recapitulating the process of CRC dissemination. CTCs derived from this model exhibit stem-cell like characteristics and are able to form colonies *in vitro* and tumors *in vivo*. Our results provide new insight into the biology of CRC-derived CTCs and may provide new therapeutic targets in the metastatic cascade of CRC.

## INTRODUCTION

Distant metastases rather than the primary tumor cause tumor-related death in over 90% of colorectal cancer (CRC) patients [1]. The biologic origin of metastasis are circulating tumor cells (CTCs), which detach from the tumor, survive in circulation, attach to the endothelium within the target organ, invade the parenchyma and form new tumors [2]. The prognostic impact of CTCs in CRC has been demonstrated repeatedly [3–12]. Although the phenotype of the clinically most relevant CTCs remains subject to debate, there is a substantial body of evidence indicating a prominent prognostic role of EpCAM^+^ CTCs in CRC [13, 11]. The capabilities required for successful metastasis formation, i.e. survival and proliferation in hostile environments such as systemic circulation and the new microenvironment of the target organ, are far beyond those of ordinary tumor cells and must therefore be acquired during the process of metastasis. However, as most distant metastases strikingly resemble the primary tumor both genetically and phenotypically, the changes a tumor cell must undergo to be able to spread to a distant organ must be transient and reversible [14–16]. Knowledge of these phenotypic alterations of CTCs may potentially provide new targets to delay or even prevent the formation of new metastases.

While in breast cancer CTCs are common and can be isolated in large numbers [17, 18], CTCs are a relatively rare finding in CRC patients [4, 19]. This makes it difficult to obtain sufficient samples for the molecular characterization of CTC in CRC. Our group has recently reported the first CTC expression profiles in human CRC, which indicate an immune-escape phenotype of otherwise mostly dormant CTCs [19]. This may explain the failure of the immune system to efficiently clear CTCs from the blood stream as well as the relative resistance of CTCs to cytotoxic therapy. However, screening of a large patient cohort was necessary to obtain a valid fraction of CTCs allowing such analyses. Additionally, the blood samples had to be obtained directly from tumor- or metastasis-draining vessels in order to increase the likelihood of CTC detection. This tremendous effort resulted in only few samples that could be analyzed by qPCR, making this kind of analysis feasible only in a few highly specialized centers.

As CTCs in human CRC are too rare to be studied adequately and, in addition, corresponding sets of tumor tissue and blood samples are difficult to obtain, the development of mouse models may offer an alternative approach [20, 21].

We here present a mouse model of metastatic colorectal cancer together with a validated workflow to capture single CTCs from the circulation that enables us to reproducibly isolate CRC-derived CTCs. As EpCAM has been shown to be a valid marker of prognostically relevant CTCs, we identified the CTCs in this mouse model based on EpCAM expression. We subsequently analyzed the expression profile of the mouse-derived CTCs by qPCR and demonstrated their colony-forming capacity *in vitro* and tumorigenicity *in vivo*.

## RESULTS

### Establishment of an orthotopic mouse model for CRC-derived CTCs

To establish a mouse model with reproducible development of distant metastases and CTCs we used NSG mice, which are highly immunocompromised and have therefore high engraftment levels even if low numbers of tumor cells are implanted [23]. As tumor cell dissemination during the implantation could obscure the results of the subsequent CTC analysis, we developed a method by which the injection of tumor cells into the cecal wall is highly controlled and reproducible as it is performed by a precision micro pump and visually controlled through a microscope. Also, to prevent leakage of the tumor cell deposit, we used a matrigel tumor cell suspension which does not leak as it solidifies at body temperature.

Three different CRC cell lines (HCT116, HT-29 and SW620) were chosen for evaluation as xenograft models. HCT116 grows at a significantly higher speed *in vitro*; therefore, we chose to inject a lower number of HCT116 tumor cells to allow the tumor to form and seed distant metastases before peritoneal dissemination or bowel obstruction occur.

To initially evaluate the cell lines for their metastatic potential in this model, 1 × 10^5^ (HCT116) or 1 × 10^6^ (HT-29, SW620) were injected into NSG mice as described above. The mice were monitored daily for signs of distress and/or tumor progression. To obtain comparable data from all mice of each group, all mice of a particular group were euthanized once > 50% of the group showed signs of distress related to tumor growth.

All mice injected with HCT116 cells developed end-stage disease within 35 days after tumor cell injection (see Table 1). Upon dissection, 5/5 animals had macroscopic metastases to the liver and microscopic metastases to the lungs (Table 1). Also, peritoneal spread and hemorrhagic ascites was noted. All animals displayed detectable EpCAM^+^ CTC in circulation. We detected both single CTCs and clusters of usually < 10 CTCs.

**Table 1:** Key measurements of the different cell lines after orthotopic injection into NSG mice

Cell line	Number of inoculated cells	Survival [d]	Tumor weight [mg]	Liver metastases	Liver weight [mg]	CTC
HCT116	1 × 10^5^	35	308 ± 17	5/5	1894 ± 30	5/5
HT-29	1 × 10^6^	42	150 ± 33 ^(**)^	4/5 ^(n.s.)^	1776 ± 91 ^(n.s.)^	0/5 ^(**)^
SW620	1 × 10^6^	67	280 ± 28 ^(n.s.)^	0/9 ^(**)^	1745 ± 115 ^(n.s.)^	0/9 ^(**)^

The day of euthanasia was chosen based on the general condition of the mice; once > 50% of mice showed signs of distress, all mice of the particular group were sacrificed in order to obtain comparable data. All statistical tests were performed vs. HCT116 as control group. One-way ANOVA with Bonferroni post-testing was used for real-valued data (tumor and liver weights), the chi-squared test was used for categorical data (incidence of liver metastases/CTC). n.s., not significant; **p < 0.01.

The mice injected with HT-29 also had vigorous tumor growth, requiring euthanasia of all mice by 42 days after tumor cell injection (Table 1). Four out of five mice in this group developed hepatic metastases. However, none of the mice had detectable CTCs. The mice in the SW620 group had considerably slower tumor growth. As several mice developed anal bleeding, the group was sacrificed at day 67 after tumor cell injection. All mice had developed local tumors, but no distant metastases or CTCs were detected (Table 1).

**Figure 1:**
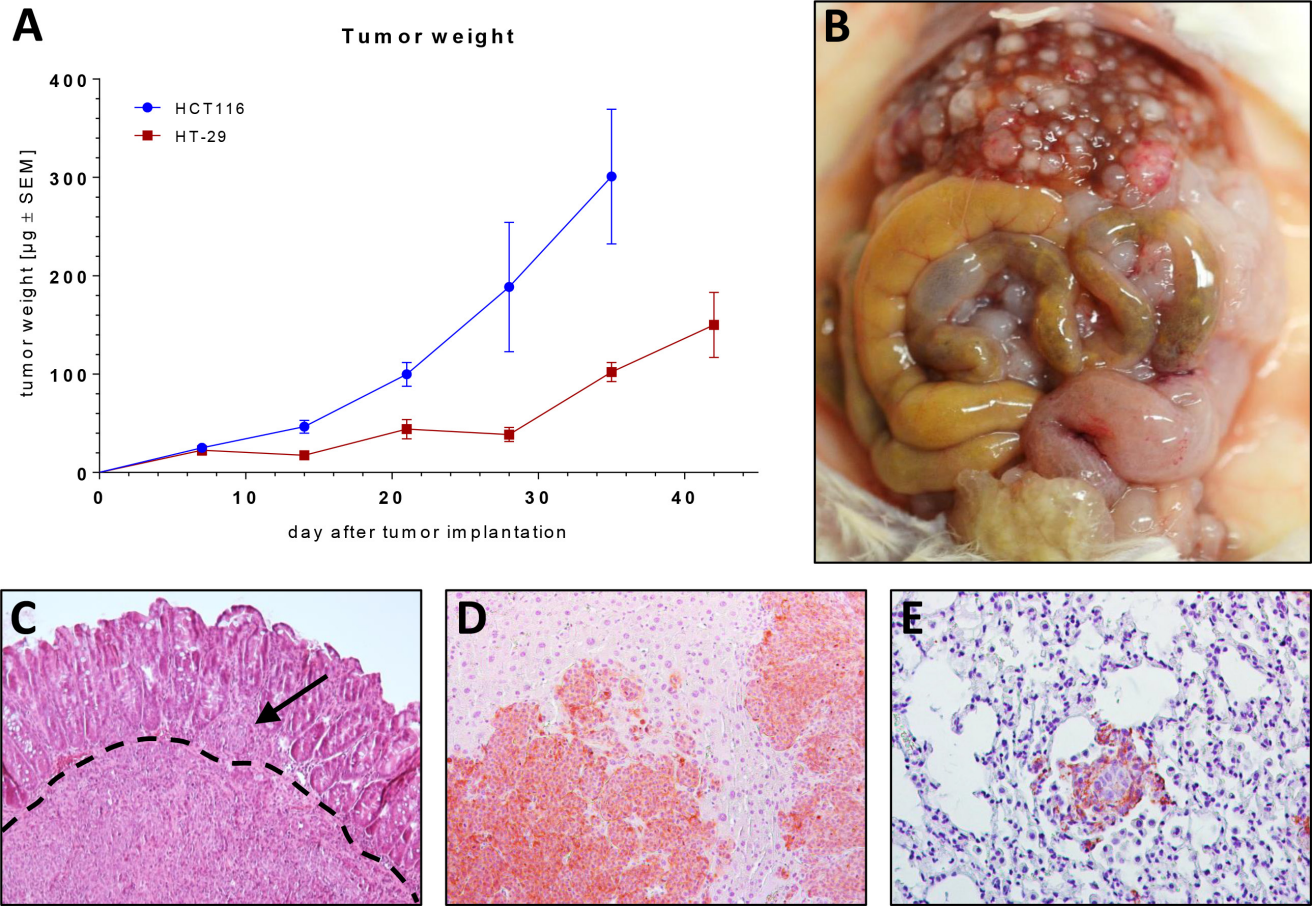
(**A**) Growth kinetics of orthotopically growing HT-29 and HCT116 tumor xenografts in NSG mice. (**B**) Situs of a NSG mouse bearing a HCT116 xenograft 35 days after tumor inoculation. (**C**) H & E section of a primary tumor (HCT116). Note the tumor bulk infiltrating the basement membrane (dashed line) and a tumor deposit on the luminal side of the basement membrane (arrow). (**D**–**E**) H & E section with immunohistochemical EpCAM staining (brown) of a liver metastasis (D) and a lung metastasis (E).

To further characterize the growth pattern of the two more malignant tumor xenografts HCT116 and HT-29, we injected 50 mice (25 / cell line) with HCT116 and HT-29 as described above. 5 mice / group were euthanized every week until day 35 (days 7, 14, 21, 28, 35), the animals were dissected and examined for local tumor growth and the development of distant metastases (Figure 1). Primary tumors, livers and lungs were sectioned and stained with H & E and EpCAM immunohistochemistry. All mice injected with HCT116 developed microscopic liver metastases until day 21 and microscopic lung metastases until day 28 after tumor cell injection. HT-29 tumors developed slightly slower and lagged about 1–2 weeks behind HCT116 tumors.

### CTCs derived from HCT116 tumors form colonies *in vitro* and tumors *in vivo*

**Figure 2:**
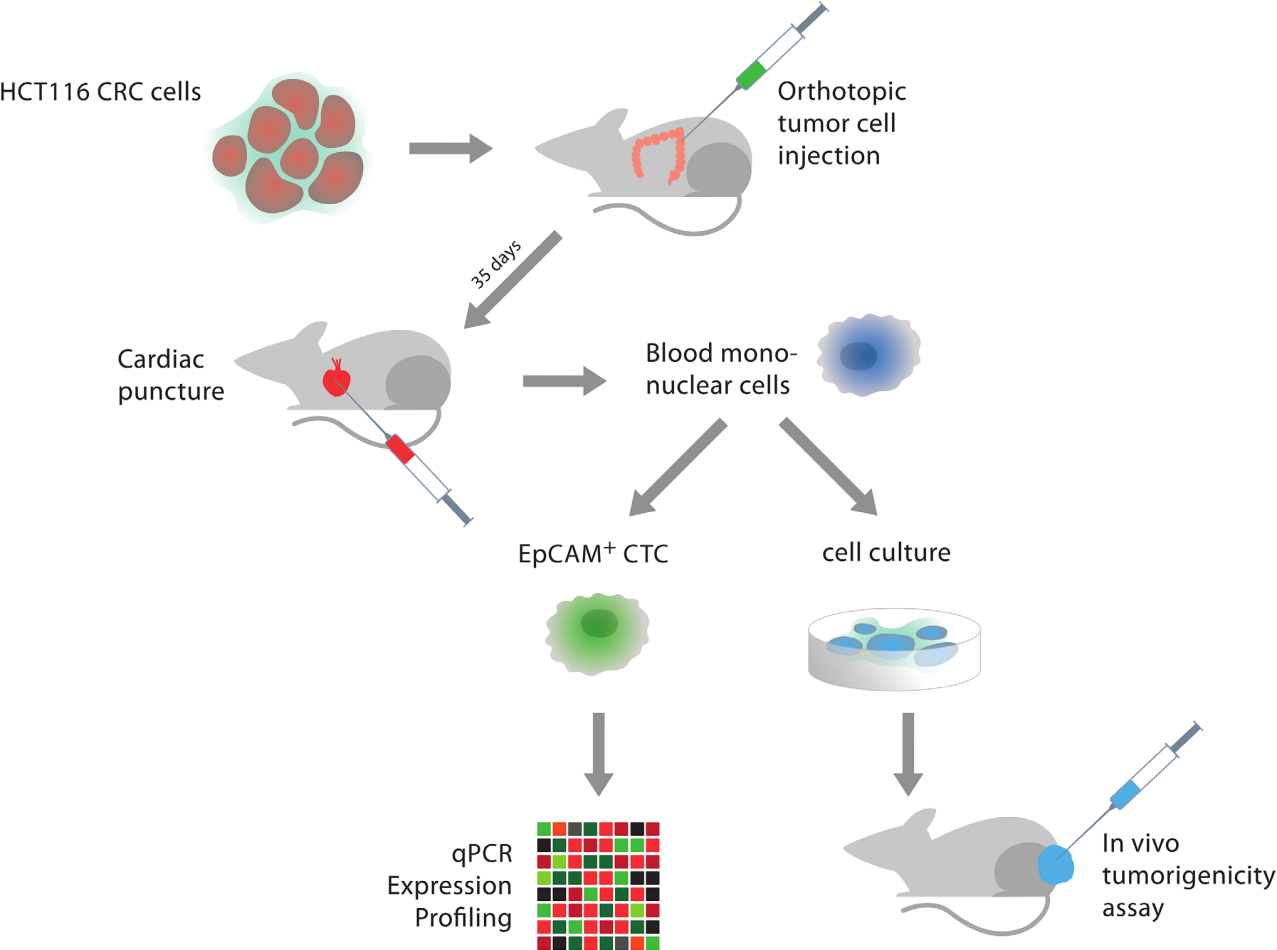
Flowchart of the experimental set-up

**Figure 3:**
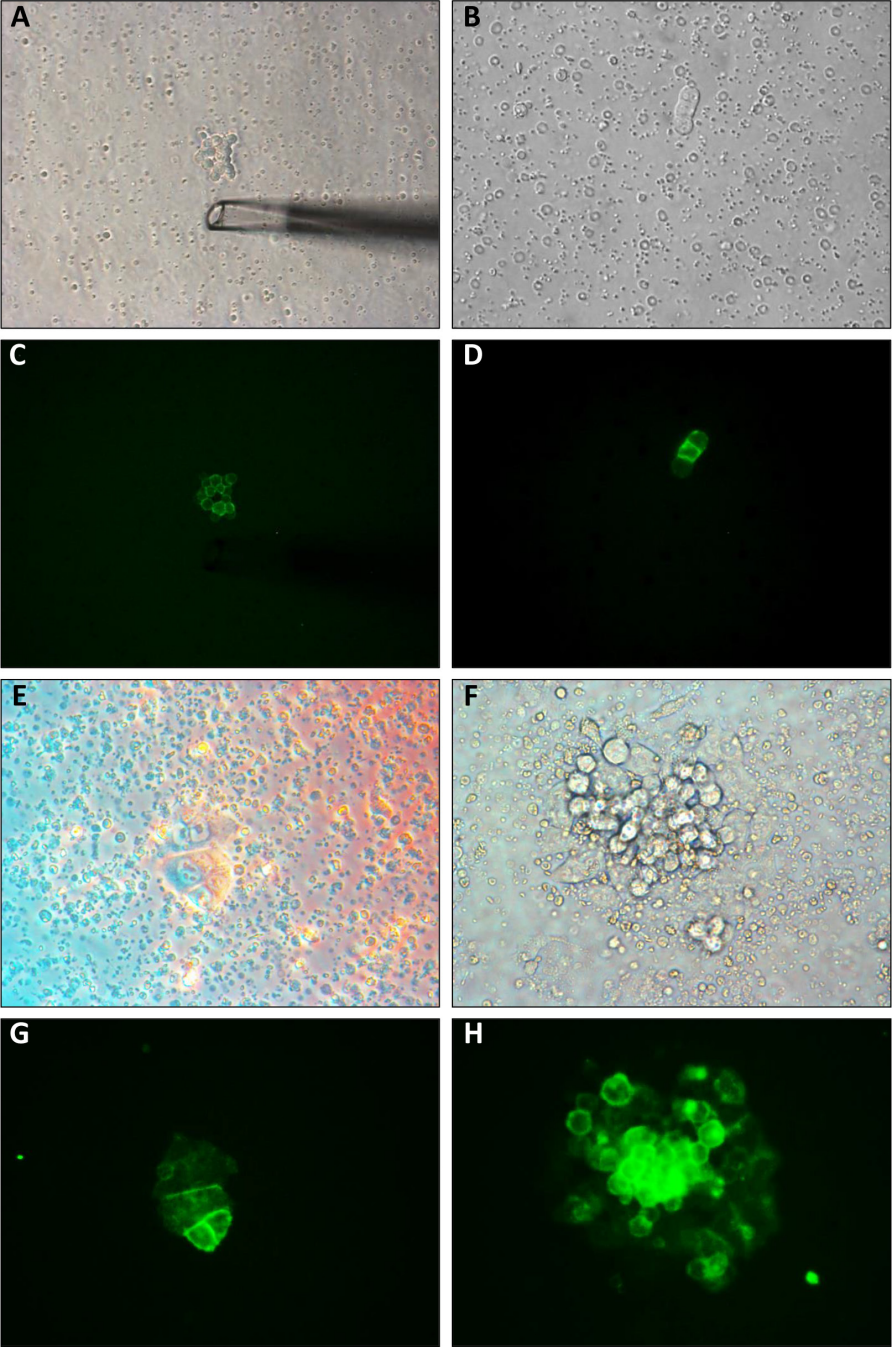
(**A–D**) CTCs among blood cells. (A–B) Native images. (C–D) Corresponding fluorescence images after staining with anti-EpCAM-Alexa 488. Note the tip of the capillary used to isolate the CTCs in image A. (**E**–**H**) Adherent colonies formed by CTCs 48 h after transfer to *in vitro* culture dishes. (E–F) Phase contrast microscopy. (G–H) Corresponding fluorescence images (anti-EpCAM-Alexa 488).

To demonstrate the tumor-forming capacity of the CTCs we orthotopically injected 5 NSG mice with HCT116 tumor cells (Figure 2). 35 days after tumor cell injection the mice were sacrificed after withdrawal of blood via cardiac puncture. BMC from the resulting 5 blood samples were isolated following a density gradient centrifugation protocol and transferred into cell culture dishes. CTCs are extremely fragile when handled *in vitro*; we therefore decided not to perform a previous EpCAM selection step. In 3 out of 5 samples, *in vitro* growth of EpCAM^+^ colonies was observed (Figure 3) and permanent CTC cell lines could be established. Upon flow cytometry analysis, ≥ 99% of cells were EpCAM^+^ (CTC: 99.43 ± 0.26% vs. HCT116: 99 ± 0.6%, Supplementary Figure S1). To demonstrate the *in vivo* tumorigenicity of these cells, 2 mice were bilaterally injected with 1 × 10^6^ cells from one of these CTC cell lines. On all (4/4) injection sites rapidly growing tumors developed, thus proving the *in vivo* tumorigenicity of the isolated CTCs.

### Expression profiling of CRC-derived CTC

As HCT116 was the only cell line that reproducibly produced CTCs in the orthotopic animal model, we subsequently used this cell line to generate tumors in 24 NSG mice. 35 days after tumor cell injection, the mice were euthanized and their blood was analyzed for CTCs. All mice had macroscopic liver metastases at necropsy. 18/24 mice (75%) had detectable CTCs (*n* = 1–250 / mL of blood, Figure 3 and Supplementary Table S1).

In the pilot experiments (see above), mice had only developed pulmonary metastases the occurrence of hepatic metastases. In addition, there is evidence that tumor-draining blood compartments (e.g., the portal vein) contain significantly more CTCs than other blood compartments [12], underlining the liver's filter effect for CTCs. We therefore hypothesized that the CTCs isolated from direct cardiac puncture are much more likely to be derived from liver metastases than from the primary tumor and compared the expression profile of CTCs to the expression of tumor cells derived from liver metastases rather than the primary tumor. As expected, the comparison of CTCs to the primary tumor produced no biologically relevant results (data not shown).

We analyzed a panel of 19 genes related to epithelial phenotype (EpCAM [*EPCAM*], *CK18*, *CK19*, *EGFR*), apoptosis (survivin [*BIRC5*]), proliferation (c-Myc [*MYC*], Ki-67 [*MKI67*]), “stemness” (DLG7 [*DLGAP5*], *BMI1*, *CD44s*, CD26 [*DPP4*]), cell migration and cell-cell contact (*CD44v6*, vimentin [*VIM*], β-catenin [*CTNNB1*], *CD151*, claudin-7 [*CLDN7*], CD166 [*ALCAM*]) and immune escape (calreticulin [*CALR*], *CD47*). To ensure robust and reproducible results and to balance out expression differences caused by individual handling of single CTCs, 5–10 individual CTCs were pooled for each CTC sample.

**Figure 4:**
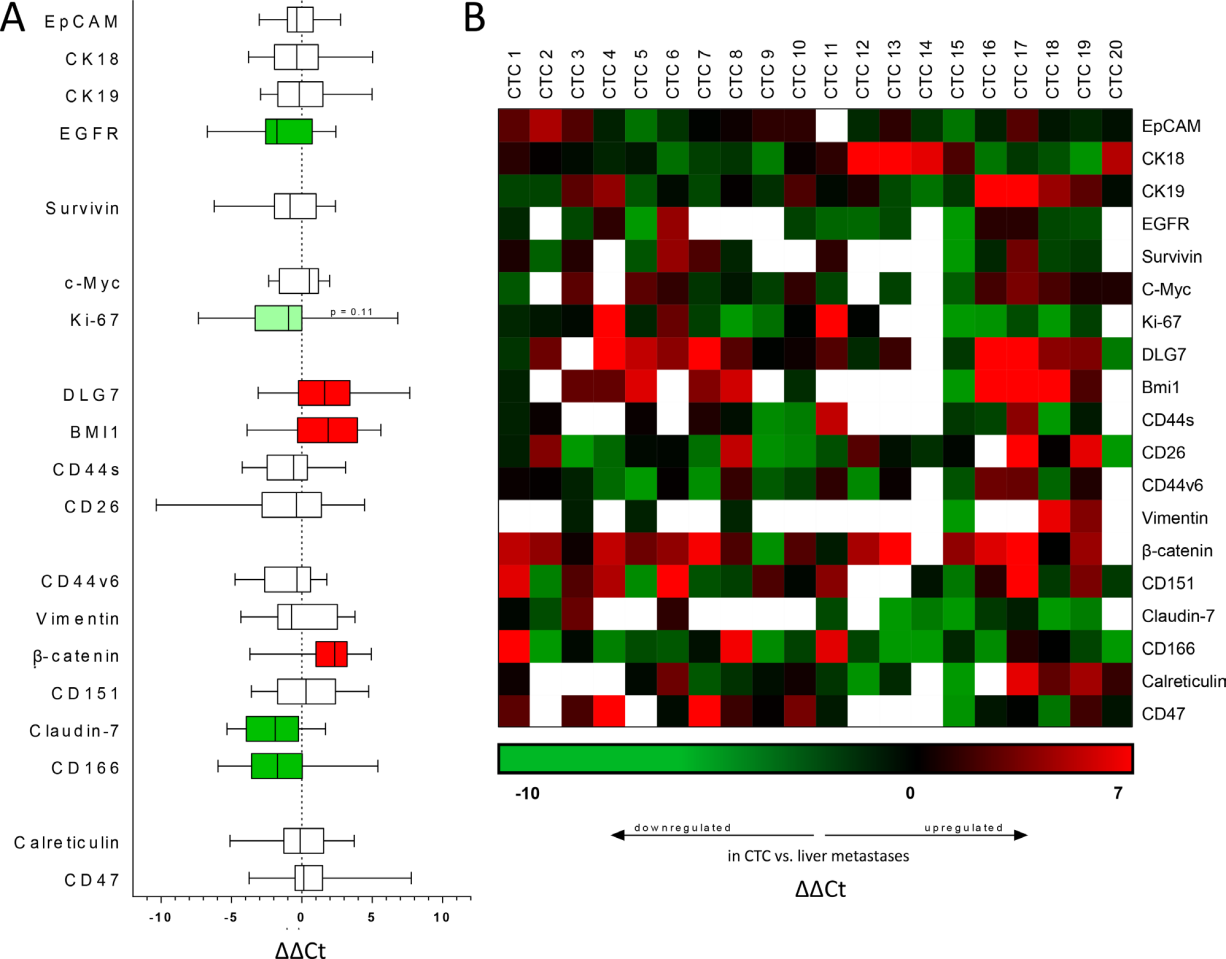
Transcriptomic analysis of CTC via qRT-PCR All data shown are ΔΔCt values comparing gene expression in circulating tumor cells vs. bulk tumor cells derived from hepatic metastases. (**A**) Gene expression of CTC compared with corresponding liver metastases. Positive values indicate increased expression, negative values reduced expression in CTC. Filled bars indicate significantly upregulated (red) or downregulated (green) genes. *p* < 0.05 was considered statistically significant. (**B**) Heat map depicting the individual ΔΔCt values of individual CTC samples compared to their respective liver metastases. Green squares indicate downregulated genes, red squares indicate upregulated genes in CTC, blank squares indicate Ct values > 35, in this case the gene was considered not expressed.

Six out of 19 genes were significantly regulated: EGFR (*p* = 0.019), claudin-7 (*p* = 0.01) and CD166 (*p* = 0.048) were downregulated, β-catenin (*p* = 0.002), BMI1 (*p* = 0.03) and DLG7 (*p* = 0.009) were upregulated. The summarized results are depicted in Figure 4A, the individual values of each sample are depicted in Figure 4B.

Generally, we observed a trend of epithelial markers (EpCAM, CK18, CK19, EGFR) to be downregulated in CTCs compared to the bulk tumor cells derived from hepatic metastases, of these genes EGFR reached statistical significance (*p* = 0.019). The expression of the anti-apoptotic protein survivin was not significantly changed. Markers of cellular proliferation tended to be downregulated in CTCs, Ki-67 was strongly downregulated but slightly failed to reach statistical significance (*p* = 0.11).

Markers generally considered to be expressed on cancer stem cells (DLG7, BMI1, CD44s, CD26) showed a strong trend towards upregulation in CTCs, 2 out of the 4 genes reached statistical significance (DLG7, *p* = 0.009; BMI1, *p* = 0.03).

Markers associated with cell-cell contact and cellular migration showed a strong tendency towards a more metastatic phenotype in CTCs; claudin-7 (*p* = 0.01) and CD166 (*p* = 0.048), both genes generally associated with cell-cell adhesion, were significantly downregulated. Conversely, β-catenin, a central hub in the canonical Wnt signaling pathway and thus closely linked to CRC progression and metastasis, was strongly overexpressed in CTCs (*p* = 0.002). In contrast to the human situation [19], no significant regulation of genes related to immune escape and immunosurveillance (calreticulin, CD47) was observed.

## DISCUSSION

Despite their clinical importance, CRC-derived CTCs are a rare biological entity and thus difficult to characterize. We here demonstrate a mouse model of CRC dissemination which enables us to reliably obtain complete sets of CTCs and corresponding tumor tissue and thus study the biology of CTCs isolated from the circulation. Three human CRC cell lines were screened for their metastatic activity when implanted orthotopically in NSG mice. HCT116 proved to be the most suitable cell line, initiating tumors that reliably metastasized to the liver and yielded CTCs in the majority of mice 35 days after tumor cell injection.

To prove the tumor-forming capacities of EpCAM^+^ cells isolated from the blood of tumor-bearing mice, we cultured CTCs without prior EpCAM selection *in vitro*. An EpCAM selection process was deliberately omitted and replaced by EpCAM screening after the initial culture step to avoid excessive handling of the CTCs and possible false-negative results. The cells were almost uniformly EpCAM^+^ in flow cytometry, formed EpCAM^+^ colonies in culture dishes and we were able to establish permanent cell lines from the *in vivo*-derived CTC colonies. Upon s.c. injection into NSG mice, the cells produced tumors on all injection sites, thereby demonstrating their tumor-forming capacity.

After demonstrating that the CTCs were in fact tumor cells, we performed qPCR expression profiling on a predefined set of genes covering biomarkers for colorectal cancer, EMT, cancer, and stemness. Several epithelial markers (EGFR, EpCAM, CK18, CK19) were downregulated in CTCs compared to the corresponding tumor tissue. This is well in line with data from human CRC-derived CTCs [19]. The proliferation marker Ki-67 was downregulated, indicating a less active and non-proliferating state of CTCs compared to bulk tumor cells, which is again in accordance with the human data.

Interestingly, two genes, DLG7 and BMI1, generally associated with a stem cell-like phenotype were strongly upregulated in CTCs. This phenotype indicates increased self-renewal and tumor-initiating capacities, a finding which is in line with our observation that CTCs are able to form new colonies in cell culture and initiate new tumors when implanted into mice. Both *in vivo* and *in vitro* colony-/tumor-forming capacities are traits that only cancer stem cells possess, therefore indicating a certain degree of “stemness” in the CTCs we isolated from this mouse model.

It is a widely accepted theory that metastatic tumor cells undergo epithelial-mesenchymal transition (EMT) and, upon completion of the metastatic process, reverse to their epithelial phenotype by undergoing mesenchymal-epithelial transition (MET) [24–26]. We observed upregulation of β-catenin, a pivotal hub of the canonical Wnt signaling pathway, which is highly involved in processes generally associated with metastasis such as cell migration and invasion [27, 28]. This upregulation of β-catenin in CTCs is in line with data from another group indicating increased β-catenin expression in metastatic tumor cells in lymph node and distant metastases of CRC [29]. Downregulation of claudin-7, a protein involved in cell-cell adhesion, also fits well into this observation, indicating a more metastatic phenotype of CTCs compared to bulk tumor cells.

Downregulation of CD166 (ALCAM) in CTC seems to be contradictory to the upregulation of DLG7 and BMI1 as all three proteins have been associated with CRC stem cells. However, all studies involving CD166 as a tumor stem cell marker have been conducted on non-disseminated tumor cells. As CTCs have a very different biology from that of bulk tumor cells, they may also exhibit different stem cell markers. Therefore, expression of CD166 may be reduced in CTCs despite their putative cancer stem cell-like character. In fact, the role of CD166 in cancer is controversial and conflicting data are available on many tumor entities [30–34]. In CRC, the data on the prognostic value of CD166 is again contradictory and may depend on the localization of CD166 within tumor cells [34, 35]. However, as CD166 is involved in cell-cell contacts and maintenance of tissue architecture, it is well conceivable that downregulation of CD166 in CTCs may contribute to a more invasive phenotype consistent with the observed downregulation of Claudin-7 and the upregulation of β-catenin in CTCs.

In a recent study, we observed an upregulation of CD47 together with a downregulation of calreticulin in human CRC-derived CTCs, which in concert constitute a phenotype indicative of CTC immune escape [19]. We were not able to reproduce these results in our mouse model; neither gene showed differential regulation in any direction. This may be explained by the model organism we used: NSG mice are the most immunodeficient mouse strain available today [23]. As there is no immunosurveillance in circulation, CTCs are not subject to Mendelian pressure for camouflage. CTCs isolated from patient samples were a selection of cells that had survived in circulation. As CD47^+^/calreticulin^−^ cells are less likely to be cleared from the blood, the cells that were finally isolated and analyzed in the patient study represented a highly selected cell population. This selection effect is not present in NSG mice, therefore we could not observe any differential regulation of CD47 or calreticulin in the isolated CTCs.

The CTC isolation process in this study was based on human EpCAM in a murine organism, eliminating the problem of non-tumor-derived EpCAM^+^ cells diluting the results. However, as our data both in patients and the mouse model strongly point toward a loss of epithelial markers including EpCAM in CTCs, it is conceivable that EpCAM^low^ or EpCAM^−^ CTCs have not been included in the analysis. This is supported by the presence of metastases despite the absence of EpCAM^+^ CTCs in the HT-29 model. On the other hand, most current studies about CRC-derived CTCs have been conducted on EpCAM^+^ CTCs and it is well accepted that these cells are prognostically highly relevant [13, 36]. The role of EpCAM^−^ CTCs is still subject to debate; however it cannot be excluded that EpCAM^−^ CTCs may display a highly malignant and clinically relevant phenotype as they have completely undergone EMT. Further studies exploring this issue are clearly warranted. New methods of CTC isolation independent of EpCAM expression are in development and clinical validation [37]; however, until today, EpCAM-based CTC detection and quantification (namely the Veridex CellSearch System) remains the FDA-approved gold standard and has proven its validity as well as prognostic and predictive value in numerous studies [3, 4, 11, 19, 12, 38, 39], making it the best option available today.

In line with the possibility of EpCAM^−^ CTCs is another limitation of this work, the isolation and culture of CTCs without a prior EpCAM selection step. We deliberately omitted EpCAM selection in the CTC used for culture and tumorigenicity assays, as in our experience CTCs are extremely fragile *in vitro*. We therefore decided to skip EpCAM enrichment prior to cultur in order to avoid false-negative results. In retrospect, this decision was supported by the fact that the CTC cell lines proved to be almost exclusively EpCAM^+^ both in culture and upon flow cytometry. Based on these methods, however, we cannot rule out the possibility that a few EpCAM^−^ CTCs have been included in the tumorigenicity assays.

In order to form metastases, CTCs have to transition from their dormant, circulating state to an actively proliferating form. *In vivo*, this supposedly happens after extravasation into the target organ. In the here presented experimental setting, the CTCs were initially seeded into culture dishes and allowed to proliferate *in vitro* before re-injection into mice. This approach was chosen to increase the robustness of the experiment and to avoid false-negative results, but may be seen as a limitation of this experiment. However, as the phenotype of the CTCs is variable and changes with the surrounding microenvironment, it is conceivable that the intermediate step of *in vitro* culture only increases the number of injected cells and does not change their ultimate *in vivo* phenotype. This hypothesis is supported by the similar histology of primary tumor, liver metastases and subcutaneous tumors formed by re-injected CTCs (data not shown).

In summary, we were able to establish an orthotopic mouse model of CRC faithfully recapitulating CTC dissemination and the process of distant metastasis in a predictable and reproducible manner. We were able to isolate CTCs from these mice, demonstrated their *in vitro* and *in vivo* tumorigenicity and performed a qPCR expression study on the CTCs, indicating stem cell properties together with reduced cell-cell adhesion in CRC-derived CTCs.

## MATERIALS AND METHODS

### Cell culture

The human CRC cell lines HCT116, HT-29 and SW620 were obtained from DSMZ (Deutsche Sammlung von Mikroorganismen und Zellkulturen; HCT116, HT-29) or ATCC (American Type Culture Collection; SW620) and cultured in standard cell culture conditions (37°C, 5% CO_2_) in DMEM (Dulbecco's modified Eagle's medium; HCT116, SW620) or RPMI (Roswell Park Memorial Institute medium; HT-29) supplemented with 10% fetal bovine serum (FBS) and 1% penicillin/streptomycin. All cell culture reagents were purchased from PAA. Regular tests for contamination were performed; the authenticity of the cell lines was regularly verified by DSMZ.

### Animal experiments

All animal experiments were independently reviewed and permitted by an institutional and a governmental Animal Care and Use Committee and were conducted according to FELASA guidelines. All possible measures to minimize suffering including anesthesia and analgesia or, if necessary, premature euthanasia were taken.

### Breeding

NOD.Cg-*Prkdc^scid^ Il2rg^tm1Wjl^*/SzJ (NOD scid gamma, NSG) breeder pairs were obtained from The Jackson Laboratory and bred in the breeding units of Interfakultäre Biomedizinische Forschungseinrichtung (IBF) of University of Heidelberg under SPF conditions. At 7–8 weeks of age, the offspring was transferred to the experimental units and enrolled in the experimental studies. Access to water and standard laboratory animal diet was granted ad libitum; the animals were housed in a temperature- and humidity-controlled environment with a 12-hour light/dark cycle.

### Orthotopic CRC model

Mice underwent general anesthesia by sevoflurane inhalation and buprenorphine injection and were placed in a supine position. Midline laparotomy was performed and the cecum was exteriorized. A syringe with a 30 G needle mounted on a microinjection pump (World Precision Instruments) on a micromanipulator (World Precision Instruments) was used to inject 1 × 10^5^ (HCT116) or 1 × 10^6^ (HT-29, SW620) CRC cells resuspended in 20 μL of matrigel (BD Biosciences) subserosally into the cecal wall. The injection was performed under direct visual control via a binocular surgical microscope. Thus, injection into or near a blood vessel which would result in direct intravascular dissemination was avoided. After injection of the cells, the abdomen was flushed to remove remaining tumor cells that may lead to artificial peritoneal dissemination and then the abdomen was closed with 6-0 PDS II (Ethicon) running sutures and surgical wound clips. After tumor induction, the mice were monitored daily for signs of distress.

### Isolation of CTCs

The isolation of CTCs has been described in detail previously [19]. Briefly, mice were anesthetized with sevoflurane as described above. About 1000 μL of whole blood were drawn via transthoracic cardiac puncture into a 1 mL syringe pre-filled with 100 μL of Na-Heparin, followed by euthanasia of the animals via cervical dislocation. The blood mononuclear cells (BMC) were then separated from erythrocytes and granulocytes by a density gradient centrifugation protocol (LSM 1077 lymphocyte gradient, PAA) according to the manufacturer's instructions. A blocking buffer (Venimmun^®^ N 1:20, Centeon Pharma) was added and the samples were stained for human epithelial cell adhesion molecule (EpCAM, anti-human EpCAM mouse IgG2b, Alexa Fluor 488, clone 9C4, Biolegend). EpCAM^+^ CTC were subsequently identified under a fluorescence microscope (Leica), picked with a micromanipulator (Eppendorf) and a glass capillary and immediately transferred into RNA lysis buffer (PicoPure RNA isolation kit, Invitrogen). For each sample, 5–10 CTCs were pooled. If more than 10 CTCs were found in a blood sample of a single mouse, several CTC specimens were created, each containing 5–10 CTCs.

### CTC tumorigenicity assay

BMC from tumor-bearing mice were obtained by density gradient centrifugation (see above), resuspended in DMEM + 10% FBS + 1% penicillin/streptomycin and transferred into cell culture dishes. 12 hours later, non-adherent cells were washed away with PBS and the adherent cells were kept in culture. Alexa Fluor 488-conjugated hEpCAM antibody (clone 9C4, Biolegend) was added and the colonies were checked for EpCAM expression, which was present in all colonies. The resulting CTC cell culture was expanded for 2 passages (1–2 weeks), after which 1 × 10^6^ cells were reinjected subcutaneously into both flanks of NSG mice. The mice were daily monitored for tumor development.

### Flow cytometry

CTC and HCT116 cell lines were harvested, stained in FACS buffer (PBS, 1% fetal bovine serum) with Alexa Fluor 488-conjugated hEpCAM antibody (clone 9C4, Biolegend) according to the manufacturer's instructions and analysed on a standard BD FACSCalibur flow cytometer (BD Biosciences). An unstained sample of each cell line was used to determine the background fluorescence and ensure proper gating, the resulting data was analyzed with WinMDI Software v2.9.

### Tumor tissue processing for real-time PCR

Analogously to previous studies [19], we compared the expression profile of CTCs with the expression profile of tumor cells derived from hepatic metastases. Tissue specimens from liver metastases were minced through a 40 μm cell strainer (BD Bioscience) and collected in PBS. The resulting cell suspension underwent the same EpCAM staining and manual isolation procedure as BMC from blood to ensure identical stress conditions on both CTCs and corresponding bulk tumor cells.

### Histology

Anmials were sacrificed by cervical dislocation, tumors, livers and lungs were removed, fixed in formalin and embedded in paraffin following standard laboratory procedures. 2 μm sections of each sample were cut and stained with hematoxylin and eosin by standard methods.

Immunohistochemical staining against human EpCAM was done with pre-labeled (Solulink All-in-One HRP conjugation kit) MOC31-HRP antibody (IQ Products) and ABC Pro Vecta Stain kit (Dako) according to the manufacturers’ instructions and standard laboratory procedures.

### RNA extraction, amplification and real-time PCR

The following procedures have been described in detail elsewhere [19]. Briefly, RNA extraction and amplification were done with the Arcturus PicoPure RNA isolation kit (Invitrogen) and the WT Ovation RNA amplification kit (Nugen) according to the manufacturers’ instructions. SYBR green real-time PCT was performed on a LightCycler (Roche) according to the manual. Primers were designed with Primer3Plus [22] and span large intronic regions to prevent the amplification of gDNA. For primer sequences please see Supplementary Table S2.

### Statistical methods

The statistical methods have been already described in detail before [19]. Ct values > 35 were considered unspecific. CTC expression data was normalized to the expression data from metastasis tissue by calculating ΔΔCt values with the following formula: ΔΔC*_t_* = ΔC*_t metastasis_* – ΔC*_t CTCs_* = *(C_t gene of interest (metastasis)_* – *C_t housekeeper (metastasis)_)* – *(C_t gene of interest (CTCs)_* – *C_t housekeeper (CTCs)_)*.

The data was tested for statistical significance using the Wilcoxon matched-pairs signed rank test, comparing the ΔC_t_ values of CTCs to the ΔC_t_ values of their corresponding liver metastasis. *p* < 0.05 was considered statistically significant. Graphpad Prism 6.01 (Graphpad Software Inc.) was used for statistical analysis and data plotting.

## SUPPLEMENTARY MATERIALS FIGURES AND TABLES


