# Supplementary material for: Circulating tumor cells exhibit stem cell characteristics in an orthotopic mouse model of colorectal cancer
Source: Oncotarget. 2016 Mar 25;7(19):27232–42. doi: 10.18632/oncotarget.8373 (PMC5053645; doi:10.18632/oncotarget.8373)
Supplement: Supplementary file 1 [file oncotarget-07-27232-s001.pdf]

Circulating tumor cells exhibit stem cell characteristics in an orthotopic mouse model of colorectal cancer

Supplementary Materials

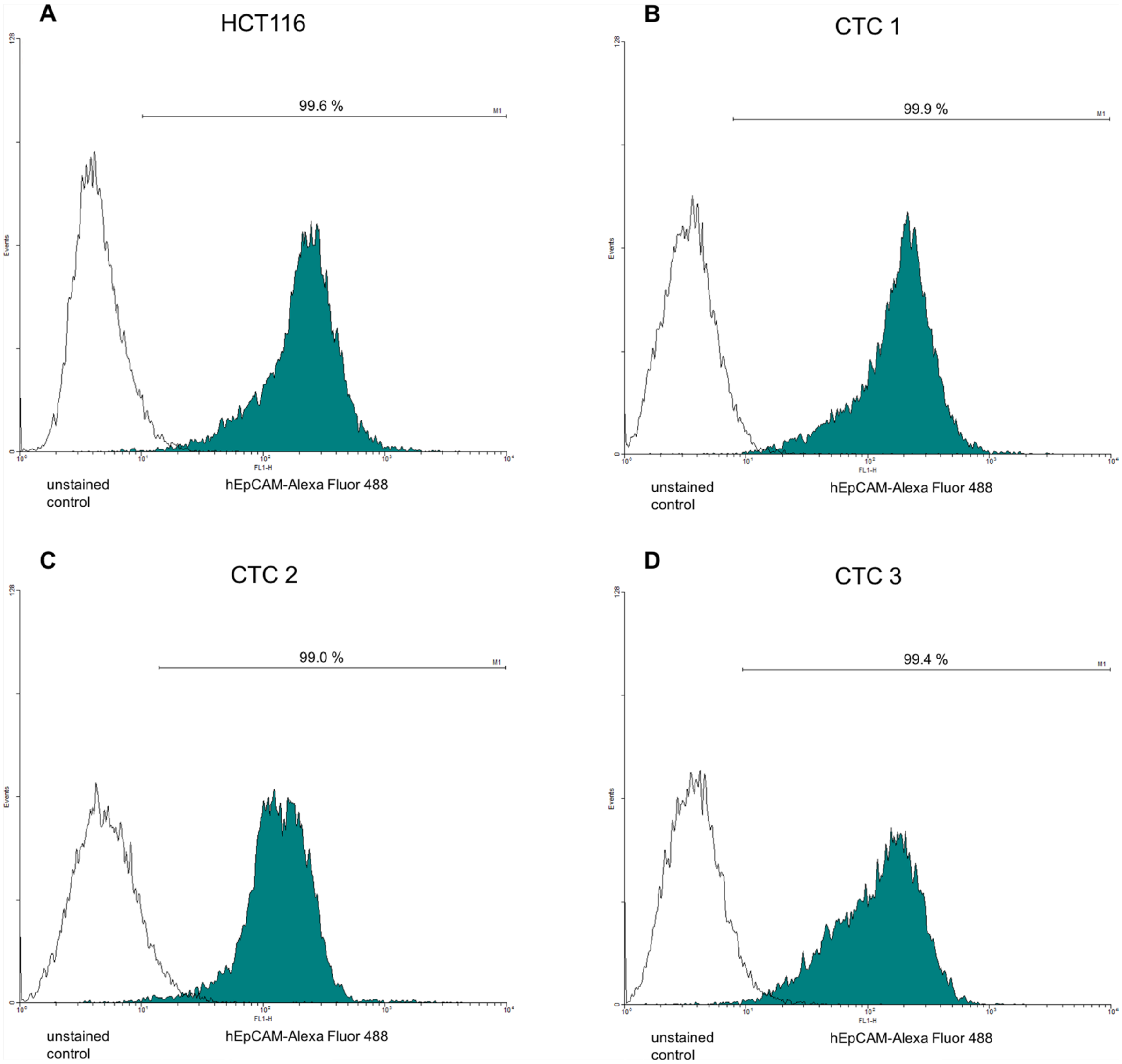

Supplementary Figure S1: Histograms of EpCAM flow cytometry of the original HCT116 cell line (A) and mouse-derived CTC cell lines (B–D). Shown are unstained (left peaks) and stained samples (right peaks). Rates of EpCAM positivity: CTC: 99.43 ± 0.26 % vs. HCT116: 99 ± 0.6 %. Data are mean ± SEM.

Supplementary Table S1: Characteristics of NSG mice 35 days after HCT116 injection

Mouse #	Tumor weight [mg]	Blood (µl)	CTC (total)	CTC/mL
1	190	1000	0	0.00
2	290	1000	2	2.00
3	340	1000	1	1.00
4	250	1000	1	1.00
5	340	900	6	6.67
6	320	950	25	26.32
7	300	550	0	0.00
8	250	500	125	250.00
9	300	1000	6	6.00
10	280	1000	10	10.00
11	220	1000	0	0.00
12	510	900	22	24.44
13	420	600	7	11.67
14	220	500	4	8.00
15	420	1000	18	18.00
16	120	1000	8	8.00
17	170	800	0	0.00
18	180	900	10	11.11
19	360	600	0	0.00
20	310	900	12	13.33
21	380	1000	2	2.00
22	340	1000	1	1.00
23	230	1000	0	0.00
24	400	1000	25	25.00

Supplementary Table S2: Primer Sequences

Target gene	Denotation	Orientation	Sequence (5′ → 3′)
CTNNB1	Bcat_2344F	Forward	TTCCGAATGTCTGAGGACAAG
CTNNB1	Bcat_2344R	Reverse	TGGGCACCAATATCAAGTCC
BMI1	Bmi1_1209Fwd	Forward	GATACTTACGATGCCCAGCAG
BMI1	Bmi-1_1209Rev	Reverse	GAAGTGGACCATTCCTTCTCC
CRT	Calr_1040F	Forward	CCACCCAGAAATTGACAACC
CRT	Calr_1040R	Reverse	TGTCAAAGATGGTGCCAGAC
PROM1	CD133_2410Fa	Forward	AAAGTGGCATCGTGCAAAC
PROM1	CD133_2410Ra	Reverse	CCGAATCCATTCGACGATAG
CD151	CD151_759Fwd	Forward	AGCAACAACTCACAGGACTGG
CD151	CD151_759Rev	Reverse	TGCTCCTGGATGAAGGTCTC
ALCAM	ALCAM3650Fwd	Forward	GAACACTGCACAGCGATTTC
ALCAM	ALCAM3650Rev	Reverse	CAAACACCAGTTTTCCTTTTCC
DPP4	CD26Fwd_2670	Forward	AGTCAGCTCAGATCTCCAAAGC
DPP4	CD26Rev_2670	Reverse	TGTGCTGTGCTGCTAGCTATTC
CD44s	CD44s_Fa	Forward	AAAGGAGCAGCACTTCAGGA

CD44s	CD44s Ra	Reverse	TGTGTCTTGGTCTCTGGTAGC
CD44v6	CD44v6_Fb2	Forward	GTACAACGGAAGAAACAGCTACC
CD44v6	CD44v6_Rb2	Reverse	TGTTGTCGAATGGGAGTCTTC
CD47	CD47_1057F	Forward	ATAGCCTATATCCTCGCTGTGG
CD47	CD47-1057R	Reverse	CGGAGTCCATCACTTCACTTC
KRT18	CK18_1288Fwd	Forward	CCCTGCTGAACATCAAGGTC
KRT18	CK18_1288Rev	Reverse	TCAGACACCACTTTGCCATC
KRT19	CK19TN_Fwd563	Forward	GCGAGCTAGAGGTGAAGATCC
KRT19	CK19TN_Rev563	Reverse	TGTCGATCTGCAGGACAATC
CLDN7	CLDN7_656Fa	Forward	TGAGCTGCAAAATGTACGACTC
CLDN7	CLDN7_656 Ra	Reverse	CACAAACATGGCCAGGAAG
MYC	C-myc_1327F	Forward	TGCTCCATGAGGAGACACC
MYC	C-myc_1327R	Reverse	GATCCAGACTCTGACCTTTTGC
CXCR4	CXCR4_1000F	Forward	CATCATGGTTGGCCTTATCC
CXCR4	CXCR4_1000R	Reverse	CGATGCTGATCCCAATGTAG
CCND1	CCND1Fwd_933	Forward	TCCTCTCCAGAGTGATCAAGTG
CCND1	CCND1Rev_933	Reverse	TTGGGGTCCATGTTCTGC
DLGAP5	DLGPA5fwd_2338	Forward	TGAAAGCAGGAGCAGCATAG
DLGAP5	DLGPA5rev_2338	Reverse	ATCTGCTACTCCACCAGCAAG
CDH1	E-Cad2563F	Forward	AGAGGACCAGGACTTTGACTTG
CDH1	E-Cad2563R	Reverse	TCAGTATCAGCCGCTTTCAG
EGFR	EGFR_3517Fwd	Forward	TTCTTGCAGCGATACAGCTC
EGFR	EGFR_3517Rev	Reverse	TGGGAACGGACTGGTTTATG
EGR1	EGR1_577F	Forward	CAGCACCTTCAACCCTCAG
EGR1	EGR1_577R	Reverse	AGCGGCCAGTATAGGTGATG
EPCAM	EpCAM_733Fa	Forward	CTGGATCCAAAATTTATCACGAG
EPCAM	EpCAM_733Ra	Reverse	GTTCCCCATTTACTGTCAGGTC
MKI67	Ki67_8625Fwd	Forward	ACAAAAGGTGCTTGAGGTCTG
MKI67	Ki67_8625Rev	Reverse	CCTTTCCCTTTCTGATTCTGC
SERPINB	SerpinB5Fwd_877	Forward	ACACCAAACCAGTGCAGATG
SERPINB	SerpinB5Rev_877	Reverse	CTGTGACAGTGACTCTGAGTTGAG
MMP7	MMP7Fwd_822	Forward	TTGGGTATGGGACATTCCTC
MMP7	MMP7Rev_822	Reverse	GAATGGATGTTCTGCCTGAAG
MSI1	Msi1Fwd_1174	Forward	CTTTGATTGCCACAGCCTTC
MSI1	MsiRev_1174	Reverse	GCTGGCTCACTCGTGGTC
CDKN1A	p21Fwd_571	Forward	ATGTGGACCTGTCACTGTCTTG
CDKN1A	p21Rev_571	Reverse	GGATTAGGGCTTCCTCTTGG
SNAI1	SnailR_680	Forward	TCTTGACATCTGAGTGGGTCTG
SNAI1	SnailFb_680	Reverse	TCTAGGCCCTGGCTGCTAC
BIRC5	Birc5Fwd_232	Forward	TTTTCATCGTCGTCCCTAGC
BIRC5	Birc5Rev_232	Reverse	AGCCCGGATGATACAAACAG
TP53	p53Fwd_1297	Forward	TGAATGAGGCCTTGGAACTC
TP53	P53Rev_1297	Reverse	TTTTATGGCGGGAGGTAGAC
VIM	Vim1483F	Forward	TTTTCCTCCCTGAACCTGAG
VIM	Vim1483R	Reverse	CGTGATGCTGAGAAGTTTCG
ACTB	b-Act_1404Fa	Forward	ATGTGGCCGAGGACTTTGATT
ACTB	b-Act_1510Ra	Reverse	AGTGGGGTGGCTTTTAGGATG
